# Modeled Aqueous Humor Protein Concentrations to Enable Biomarker Development in Uveal Melanoma

**DOI:** 10.3390/ijms27073124

**Published:** 2026-03-30

**Authors:** Elaine Huang, Yilin Chen, Chen-Ching Peng, Donny Liang, Mark Reid, Atrey Khoche, Peter Kuhn, Jeremy Mason, Xuejuan Jiang, Jesse L. Berry, Liya Xu

**Affiliations:** 1 The Vision Center at Children’s Hospital Los Angeles, Los Angeles, CA 90027, USA; elainejh@usc.edu (E.H.); ppeng@chla.usc.edu (C.-C.P.); mreid@chla.usc.edu (M.R.); khoche@usc.edu (A.K.);; 2 USC Roski Eye Institute, Keck School of Medicine, University of Southern California, Los Angeles, CA 90033, USA; xuejuanj@usc.edu; 3 USC Michelson Center for Convergent Biosciences, Department of Biological Sciences, University of Southern California, Los Angeles, CA 90089, USA; ethenche@usc.edu (Y.C.);; 4 The Saban Research Institute, Children’s Hospital Los Angeles, Los Angeles, CA 90027, USA; 5 Bridge Institute, University of Southern California, Los Angeles, CA 90089, USA; 6 Norris Comprehensive Cancer Center, Keck School of Medicine, University of Southern California, Los Angeles, CA 90033, USA

**Keywords:** uveal melanoma, aqueous humor proteomics, protein concentration modeling

## Abstract

Uveal melanoma (UM) lacks minimally invasive and reproducible biomarkers to support clinical risk stratification, motivating the need for molecular profiling of aqueous humor (AH) as an alternative to fine-needle tumor aspiration (FNAB). This study aimed to generate a calibrated AH protein concentration map to identify tumor-associated signals present at clinically measurable levels and assess their associations with established molecular and clinical features. AH samples from 70 UM eyes were analyzed using next-generation sequencing-based proximity extension assays (PEAs), and leftover AH from 27 samples was further assessed using qPCR-based PEA to obtain reference concentration values. Regression models derived from overlapping proteins enabled extrapolation of calibrated pg/mL-level concentrations across the full cohort. Twenty-three proteins had median modeled concentrations above 5 pg/mL and were examined for clinical relevance and translational feasibility. Several proteins, including CXCL8, CXCL10, VEGFA, HGF, PDCD1, FLT1, FLT3LG, and CCL2, showed progressive increases from GEP1/PRAME− to GEP2/PRAME+ tumors and from AJCC Stage I/II to Stage III/IV, with Stage IV tumors demonstrating significant elevations in CXCL8, VEGFA, and PDCD1. Pathway analysis revealed activation of inflammatory and tumor microenvironment pathways, and upstream regulator analysis identified VEGFA and CCL2 as potential drivers. These findings demonstrate that calibrated AH proteomic profiling can identify clinically measurable protein changes associated with UM risk and stage, supporting its potential utility for biomarker development.

## 1. Introduction

Uveal melanoma (UM) is the most common primary intraocular malignancy in adults [1] and carries a high risk of metastasis, primarily to the liver, with up to 50% of patients developing distant disease despite effective local control [2]. Because metastatic UM remains largely incurable, there is a critical need for early, accurate, and minimally invasive tests to guide surveillance and management [3,4,5]. Current prognostic stratification relies on fine-needle aspiration biopsy (FNAB) for gene expression profiling (GEP) [6] and assessment of the Preferentially Expressed Antigen in Melanoma (PRAME) status [7,8], which together inform metastasis risk. Although effective, FNA requires specialized expertise and carries procedural risks that can limit its use, and in real-world practice, a substantial proportion of UM eyes—including over 30% in our cohort—do not undergo biopsy due to clinical contraindications, tumor characteristics, or patient preference [9,10,11].

Aqueous humor (AH) biopsy has emerged as a minimally invasive alternative, enabling molecular profiling of the intraocular tumor environment and tumor-associated signals when FNAB is not performed [12]. Proteomics-based liquid biopsy approaches utilizing both vitreous and aqueous humor have demonstrated promise in identifying biomarkers associated with UM progression and metastasis risk [13,14,15]. However, to date, most AH proteomic studies have relied on relative quantification metrics, such as normalized expression (NPX) values, two-dimensional gel electrophoresis, and novel flow-cytometry-based approaches, without estimating absolute protein concentrations in units that support clinical translation [12,14,16]. Relative metrics are valuable for discovery, but without calibrated concentration units, they do not establish whether a signal is present at levels compatible with clinically deployable assays, limiting reproducibility, cross-study comparisons, and the development of diagnostic thresholds. Focusing on proteins present above practical detection limits—such as those measurable by standard immunoassays—is essential for advancing clinically deployable biomarkers. Because AH reflects an integrated ocular compartment, this study focuses on identifying tumor-associated signals that reach AH at concentrations compatible with clinical testing. Prior analyses have largely relied on small, predefined cytokine panels, which limit translational applicability by pre-selecting targets without establishing whether those proteins are present in aqueous humor at concentrations compatible with clinical assay development [15,17,18].

To address these gaps, we used proximity extension assays (PEAs) combined with regression-based calibration to estimate absolute protein concentrations (pg/mL) in AH from patients with UM. Our goal was to generate a calibrated molecular map of the AH protein targets and characterize how these concentrations relate to established prognostic and clinical features. Establishing calibrated protein concentration ranges may support the development of reproducible diagnostic thresholds and facilitate future translational studies using AH as a minimally invasive platform [19] for UM risk stratification and longitudinal monitoring.

## 2. Results

### 2.1. Clinical Demographics

**Figure 1:**
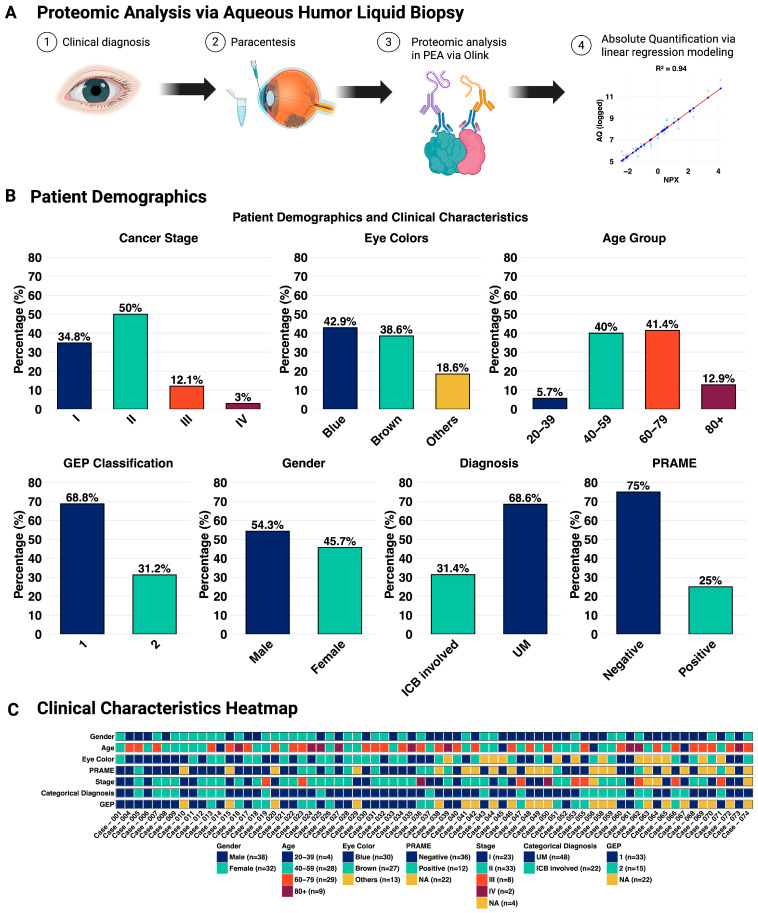
Demographic and clinical characteristics. (**A**) Overview of study design, including sample collection, (**B**) patient demographics, and (**C**) heatmap of clinical characteristics. The patient cohort includes samples from 33 GEP1 (low-risk) and 15 GEP2 (high-risk) UM patients, all used for subsequent proteomic analysis. Created in BioRender. Huang, E. (2026) https://biorender.com/t4bewne, accessed on 8 February 2026.

A total of 70 AH samples were collected from 70 diagnostic UM eyes at the time of plaque brachytherapy placement. Of these, GEP of tumor FNAB classified 33 samples as GEP1 (low metastatic risk) and 15 samples as GEP2 (high metastatic risk). Biopsies were not performed in 22 cases. Demographic and clinical characteristics of the cohort are shown in Figure 1. The cohort included 38 male (54.3%) and 32 female (45.7%) patients, with a median age at diagnosis of 61 years (range: 22–97 years). Uveal melanoma with choroidal involvement was present in 48 eyes (68.6%), while 22 eyes exhibited uveal melanoma involving the iris or ciliary body (ICB) (31.4%). Among the eyes that underwent biopsy, 75% of eyes were PRAME-negative, while 25% were PRAME-positive. Eye color distribution showed a predominance of blue eyes (42.9%), followed by brown (38.6%) and others (18.6%).

### 2.2. Generating Calibrated Protein Concentration Estimates

**Figure 2:**
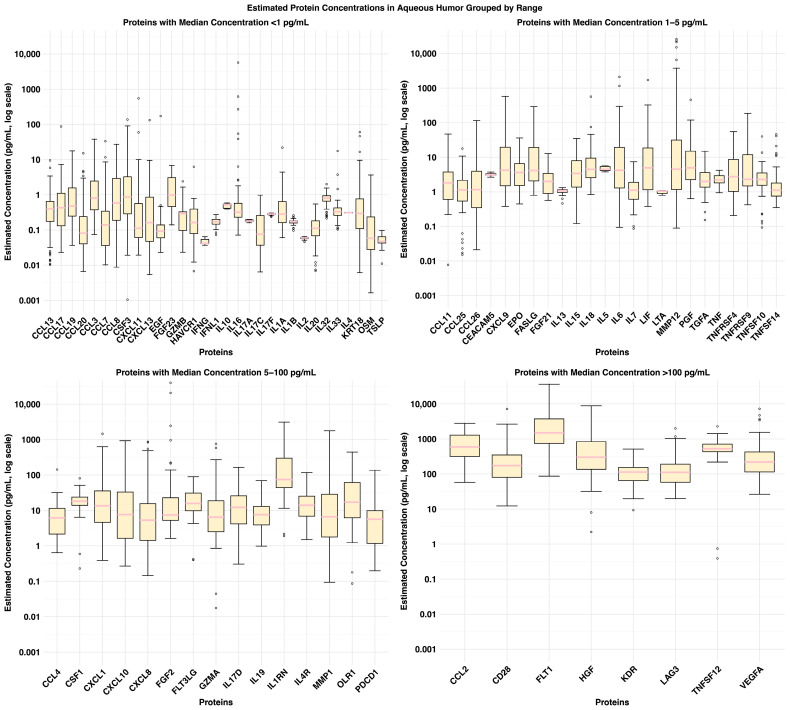
Estimated Concentration Range of Key Proteins. Boxplots depicting absolute protein concentrations (pg/mL, log10 scale) grouped by median concentration range. Each panel represents a different concentration range, with individual proteins plotted within each group. Concentration variability within each protein is represented by the interquartile range, median, and whiskers (1.5× IQR), with outliers shown as individual points.

**Figure A1:**
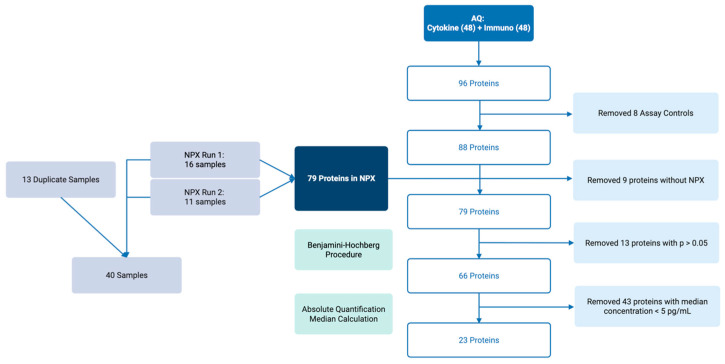
Methodology, workflow, and sample collection. Created in BioRender. Huang, E. (2026) https://biorender.com/t4bewne, accessed on 8 February 2026.

**Figure A2:**
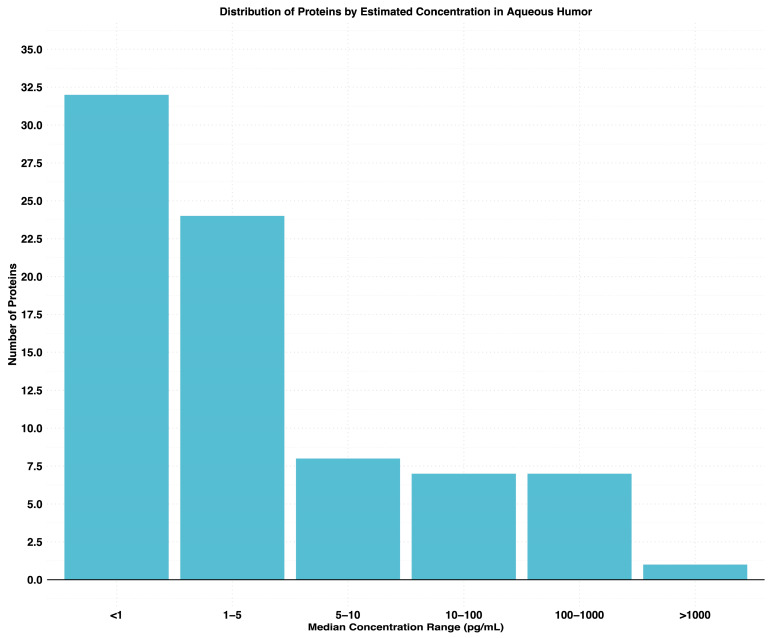
Distribution of absolute concentrations (pg/mL) for key proteins across all aqueous humor samples.

Using a dual-platform proximity extension assay (PEA), we generated calibrated protein concentration estimates (pg/mL) across 79 proteins in AH from the full 70-sample cohort (Figure A1). Protein concentrations ranged widely, from sub-picogram levels to nanograms per milliliter (ng/mL). The distribution of absolute concentrations for 79 proteins across all aqueous humor samples is listed (Figure A2). Among them, 23 proteins exhibited median calibrated concentrations above the conservative detection threshold of 5 pg/mL (Figure 2). This threshold reflects the low detection limits typical of standard immunoassays. The majority of proteins were found in the lower concentration ranges, with the highest frequency observed in the <1 pg/mL and 1–5 pg/mL ranges. A single protein, FLT1, exhibited a median estimated concentration >1000 pg/mL, distinguishing it as the most abundant among the profiled analytes (Figure 2).

**Figure 3:**
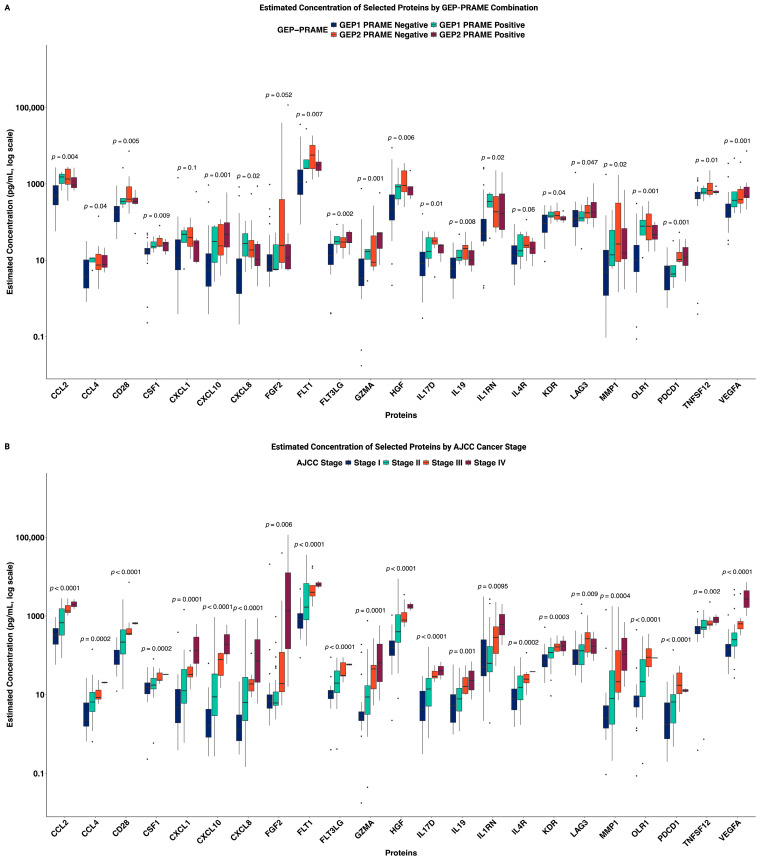
(**A**) Estimated concentration of selected proteins by GEP-PRAME classification. (**B**) Estimated concentration of differentially expressed proteins by stage. Created in BioRender. Huang, E. (2026) https://biorender.com/t4bewne, accessed on 8 February 2026.

We then focused on these 23 proteins with modeled median concentrations above 5 pg/mL, identifying them as abundant proteins in AH. The concentrations of these 23 proteins were compared among four molecular subgroups defined by GEP class (Class 1 vs. Class 2) and PRAME status (negative vs. positive; Figure 3A). Several proteins demonstrated a clear trend across prognostic categories, particularly for: GZMA, CXCL10, FLT3LG, CD28, VEGFA, HGF, and TNFSF12 (Figure 3A). As expected, cases with GEP1 and PRAME negativity (*n* = 28) had the lowest concentration of these proteins, correlating with improved prognosis and less severity of disease. These comparisons reflect cross-sectional associations and not longitudinal progression.

We subsequently evaluated the calibrated concentrations of these proteins above the detection threshold across AJCC clinical stages (Figure 3B; Stages I to IV). Higher concentrations were observed in later-stage tumors for multiple proteins, including CXCL8, CXCL10, CCL4, GZMA, MMP1, IL17D, CXCL1, IL19, IL4R, FLT3LG, CSF1, IL1RN, KDR, CD28, VEGFA, HGF, CCL2, TNFSF12, and FLT1, with generally increasing levels from minor (Stages I/II) to advanced (Stages III/IV) stages.

**Figure A3:**
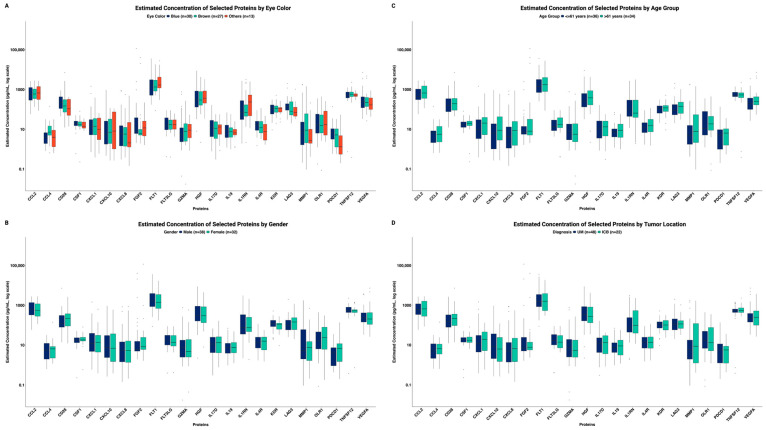
Estimated concentration of 23 selected proteins by (**A**) eye color, (**B**) gender, (**C**) age group, and (**D**) tumor location.Created in Created in BioRender. Huang, E. (2026) https://biorender.com/t4bewne, accessed on 8 February 2026.

Several proteins showed statistically significant increases in concentrations across advancing AJCC stages, including CXCL8 (*p* < 0.0001), VEGFA (*p* < 0.0001), and PDCD1 (*p* < 0.0001). Stage IV tumors (*n* = 2) showed higher median concentrations for these proteins relative to earlier stages. These patterns are consistent with enrichment of pro-inflammatory or immunoregulatory factors in more advanced disease (Figure 3B). Non-significant differences were observed when comparing eye color, age, tumor location, and gender (Figure A3). All significance values reflect rank-based tests with modeled concentration inputs. Key stage-associated proteins retained statistical significance across repeated simulations incorporating model prediction intervals, supporting robustness of the modeled estimates (Figure A5).

### 2.3. Pathway Analysis

**Figure 4:**
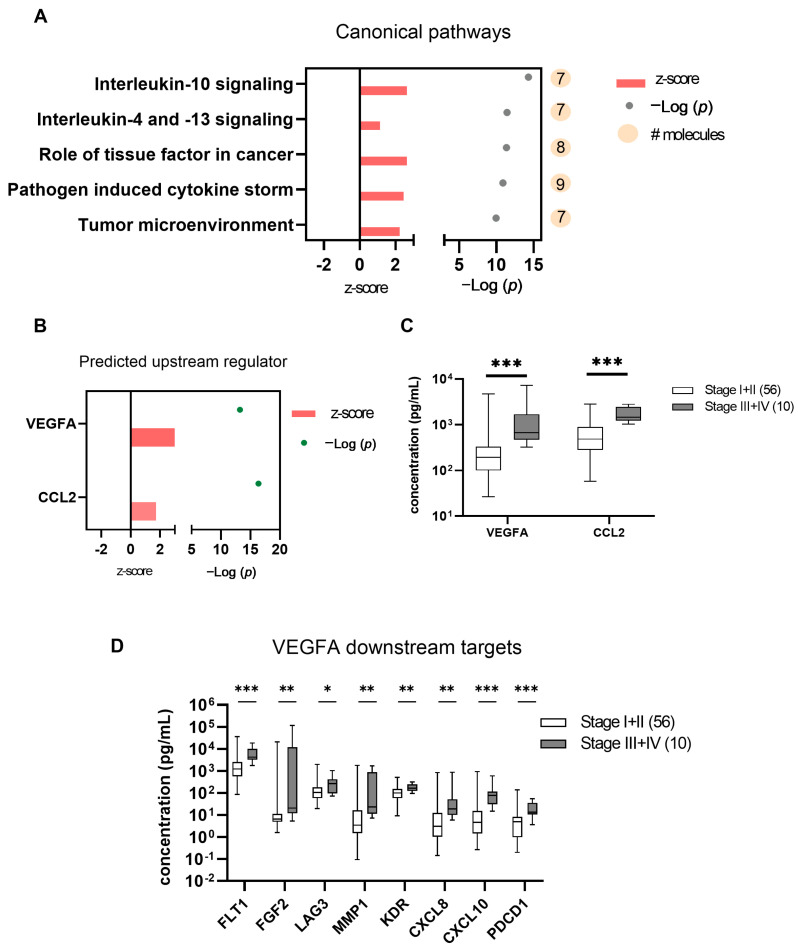
Enrichment of inflammatory and tumor microenvironment signaling of aqueous humor proteins in advanced-stage uveal melanoma. (**A**) Canonical pathway analysis of aqueous humor proteomics comparing AJCC Stage I–II and Stage III–IV tumors reveals the enrichment of inflammatory and tumor microenvironment-related pathways. Bars indicate activation z-scores, dots represent −log (*p*-value), and circle size reflects the number of molecules per pathway. (**B**) Upstream regulator analysis predicts the activation of VEGFA and *CCL2*. Red bars represent activation z-scores, and dots indicate −log (*p*-value). (**C**) Estimated concentrations of aqueous humor VEGFA and CCL2 are compared between Stage III–IV and Stage I–II tumors. Concentrations are presented on a logarithmic scale. (**D**) Expression levels of selected *VEGFA* downstream targets (FLT1, FGF2, LAG3, MMP1, KDR, CXCL8, CXCL10, and PDCD1) are elevated in advanced-stage disease, consistent with the activation of angiogenic and immune-modulatory signaling networks. Estimated concentrations are shown on a logarithmic scale. Statistical significance is indicated as * *p* < 0.05, ** *p* < 0.01, and *** *p* < 0.001.

To determine the biological relevance of the 23 proteins above the detection threshold, pathway analysis was conducted to identify the important cellular functions these DEPs may provide. After subjecting the 23 DEPs to Qiagen Ingenuity Pathway Analysis (IPA), the canonical pathway analysis revealed significant enrichment in immune and tumor-related pathways, with the top hits including Interleukin-10 signaling, pathogen-induced cytokine storm, and tumor microenvironment. These pathways exhibited positive activation z-scores, consistent with potential activation in advanced-stage tumors (Figure 4A; z-score > 2).

We subsequently performed an upstream regulator analysis (URA) to identify the potential upstream target that has been experimentally verified to affect the expression of the 23 DEPs. URA identified VEGFA (z = 3.1) and CCL2 (z = 1.7) as the strongest predicted upstream drivers (Figure 4B). Both exhibited higher expression in advanced disease stages (III–IV) compared to early stages (I–II) in AH (Figure 4C).

Evaluation of VEGFA downstream targets (FLT1, FGF2, LAG3, MMP1, KDR, CXCL8, CXCL10, and PDCD1) showed significantly higher concentrations in advanced-stage tumors, consistent with increased activity of the VEGFA-associated signaling axis in advanced disease (Figure 4D).

## 3. Discussion

This study presents the first broad-scale calibrated estimation of AH proteins in UM. Our findings demonstrate that AH proteomics can serve as a minimally invasive technique that offers greater insight into clinical characteristics of uveal melanoma, offering a safer and repeatable platform for risk stratification. Previous AH proteomics studies have primarily relied on relative expression metrics, such as NPX values, which are suitable for discovery but limited in reproducibility across cohorts and in defining clinical thresholds. By generating calibrated pg/mL estimates based on regression models, we enable standardized measurement across patients and cohorts, supporting the development of reproducible diagnostic thresholds and assays, and distinguishing proteins that are realistically measurable using conventional clinical assays.

Statistical analysis confirmed the significance of these biomarkers in distinguishing UM clinical features. The activation of immune- and tumor-related pathways in advanced-stage tumors points to a coordinated inflammatory and angiogenic response during disease progression. VEGFA and CCL2 emerged as central upstream regulators, consistent with influencing the observed proteomic shifts through vascular remodeling and immune modulation.

VEGFA, a key pro-angiogenic factor, drives tumor vascularization and may facilitate metastatic spread. It has long been established in uveal melanoma that elevated aqueous VEGFA levels correlate with tumor size, disease progression, and patient survival [20]. Hypoxia within the tumor microenvironment induces VEGFA expression via the HIF-1a pathway, suggesting that VEGFA may contribute to tumor vascularization and could serve as a therapeutic target [21]. FLT1 encodes VEGFR-1, a receptor for VEGFA, and plays a role in angiogenesis. While specific studies on FLT1 in UM are limited, its interaction with VEGFA suggests that FLT1 may be involved in tumor vascularization and progression. Targeting the VEGFA-FLT1 axis could be a potential therapeutic strategy in UM. The consistent upregulation of VEGFA downstream effectors reinforces its pivotal role, consistent with greater activity of the VEGFA-associated pathway during UM progression.

In UM, the chemokine CCL2 is produced by tumor cells and monocytes, attracting tumor-associated macrophages that can promote tumor growth and suppress immune response. Its expression is associated with chromosome 3 loss, a marker of poor prognosis in UM [17]. Elevated CCL2 levels have been reported in patients with melanoma and are associated with tumor metastasis, immunosuppression, and disease progression, as it mediates the invasion and growth of metastatic melanoma. The involvement of the CCR2/CCL2 axis is of particular importance in the early stages of tumor invasion and is regarded as a predictor of poor prognosis in cancer patients [22]. Additionally, CXCL10 facilitates the recruitment of immune cells and directly promotes tumor vascularization and growth, upregulating pro-angiogenic factors such as VEGF, PDGF-B, and FGF2 [23]. The associations observed in our AH dataset are consistent with these described mechanisms, although causal or cellular source inferences cannot be inferred from this cross-sectional analysis. Of note, pathway enrichments should be interpreted as compartment-level signals. While it is biologically plausible that these inflammatory signatures arise from a combination of tumor-intrinsic signaling and host immune responses within the ocular compartment, disentangling these contributions was beyond the scope and primary objective of the present study.

Collectively, these findings highlight distinct protein expression patterns in AH associated with both molecular features (GEP and PRAME status) and clinical progression, suggesting potential utility for stratifying disease aggressiveness in uveal melanoma. Importantly, these results do not represent a finalized diagnostic or prognostic classifier, and additional validation will be required. Rather, they demonstrate the feasibility of AH proteomics and its ability to provide calibrated clinically measurable biomarkers with both diagnostic and prognostic relevance that may support future studies. All protein concentrations represent regression-modeled estimates derived from measured reference values.

**Figure A4:**
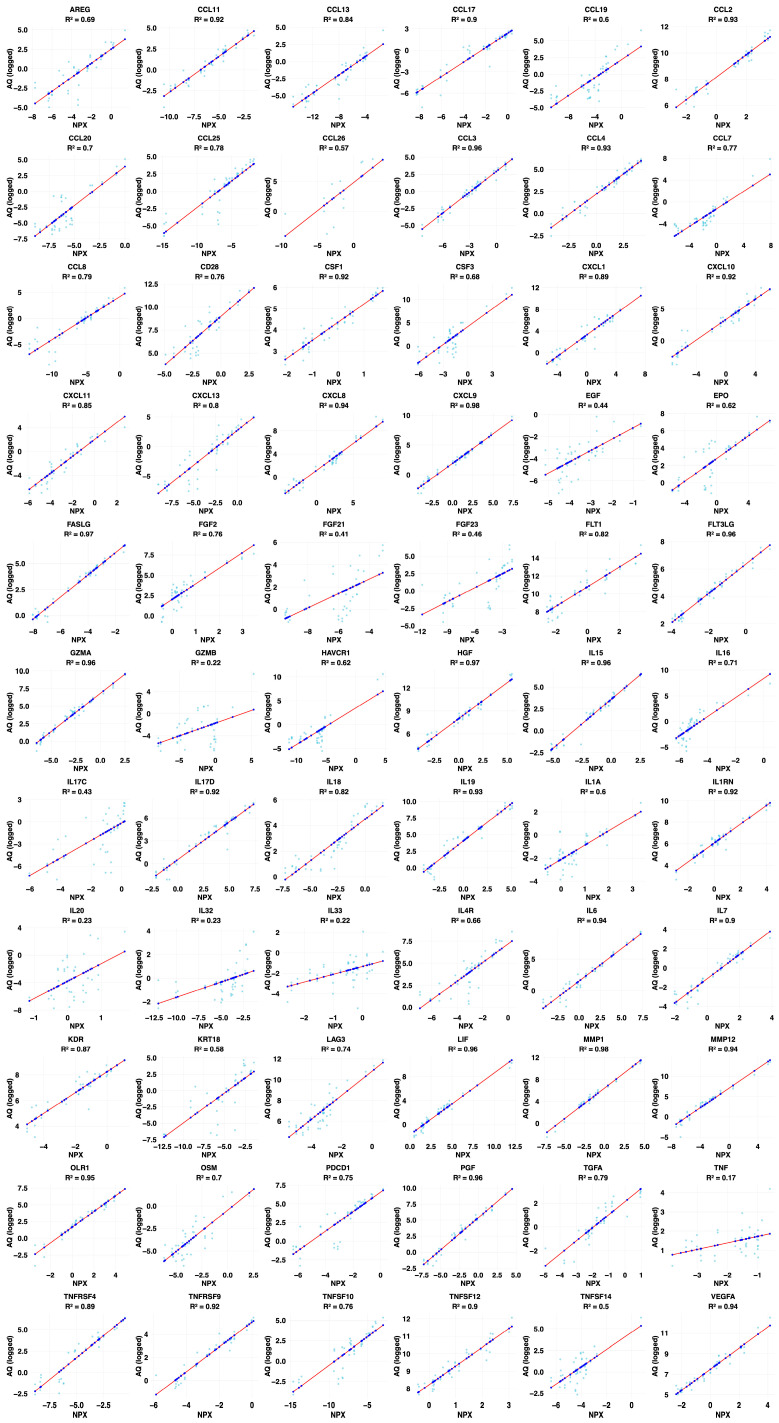
Regression plots for 66 proteins.

**Figure A5:**
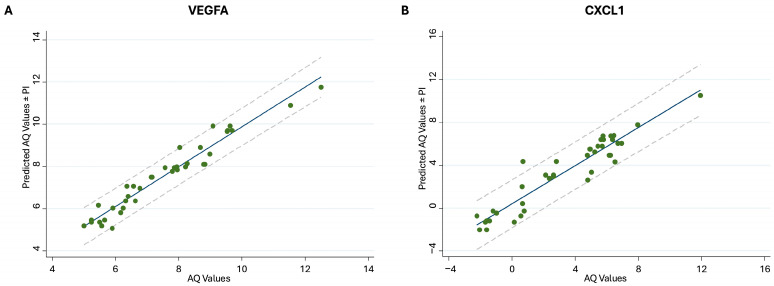
Data simulation (*n* = 100) for (**A**) VEGFA, *p* < 0.0001, and (**B**) CXCL1, *p* = 0.0001. Dashed lines indicate the prediction interval, and dots represent observed AQ values plotted against the fitted prediction line.

To address volume limitations in AH samples and enable calibrated estimation across the full cohort, we applied a calibration approach based on regression modeling (Figure A4). Reference concentrations were experimentally measured in a subset of samples and then extrapolated across the full dataset using normalized NPX values. This method allowed us to derive pg/mL values across 66 proteins while correcting for batch effects and inter-run variability. By reporting calibrated concentrations rather than relative metrics, our analysis improves reproducibility, supports cross-study benchmarking, and enables diagnostic assay development. Simulation-based sensitivity analyses incorporating prediction intervals indicated that the strongest stage-associated signals were robust to re-introduction of modeled variance, supporting the reliability of the regression-based concentration estimates (Figure A5).

Several limitations should be acknowledged. Not all AH samples yielded sufficient volume for direct quantification; qPCR-based measurements were obtained directly in only a subset (27 samples, including 13 duplicates), with extrapolation applied to the remainder. Statistical comparisons were performed using median concentrations because of high variability and non-normal distributions in this small cohort. Interpretation of stage-associated differences, particularly for Stage IV, is limited by small sample size. Although these differences remained statistically significant after FDR adjustment, preliminary analysis of samples by stage remains exploratory in nature and warrants validation in larger, multi-institutional cohorts. Additionally, the regression-based extrapolation inherently reduces variability and may produce values that appear cleaner than the true underlying biological spread.

Lastly, the extraction of AH via paracentesis is inherently procedure-dependent. It is conceivable that certain proteins measured in AH, including VEGFA, may also be detectable in less invasive biofluids, such as tears or serum [24,25]. However, compared with tears, which are influenced by environmental exposure, lacrimal physiology, and ocular surface inflammation, AH more directly reflects intraocular tumor-associated signaling. Systemic biofluids such as plasma may introduce additional confounding from systemic inflammatory or oncologic processes and may dilute tumor-derived signals below detection thresholds [26,27]. Accordingly, while distal biofluids hold translational promise, AH currently provides the most proximate and biologically interpretable fluid compartment for characterizing the uveal melanoma microenvironment.

The progressive trends observed across molecular risk groups and AJCC stages support the internal consistency and biological plausibility of the calibrated measurements; however, these findings should not be interpreted as evidence of causal or independent associations. Our current cohort from a single center was not designed or statistically powered to separate the effects of tumor size, anatomical location, AJCC stage, and molecular classification. Addressing these questions will require larger cohorts across multiple centers with predefined stratification and formal multivariable modeling. Future studies with larger, balanced cohorts and longitudinal sampling will be essential to orthogonally validate these findings using ELISA and determine their applicability for disease monitoring and clinical decision-making. Because such validation must be conducted on a protein-by-protein basis, the present study was necessary to first define a technically measurable and biologically plausible candidate set.

In summary, this study demonstrates the feasibility of AH proteomics with calibrated protein quantification, identifying a panel of proteins consistently measurable above practical thresholds that correlate with both molecular risk and clinical stage. These findings establish a foundation for future biomarker validation and assay development, advancing the translational potential of AH as a minimally invasive liquid biopsy platform in uveal melanoma.

## 4. Materials and Methods

This investigation was a case series study at a tertiary care hospital (Roski Eye Institute, University of Southern California, Los Angeles, CA, USA) conducted with approval by the Institutional Review Board (HS-19-00293). Samples were collected between 2019 and 2024.

### 4.1. Patient Clinical Characteristics and Demographics

This study included a convenience sample of 70 UM patients at the Roski Eye Institute, University of Southern California, from whom written informed consent for an AH sample was obtained. Patient demographics and clinical characteristics are summarized in Figure 1. All samples consisted of approximately 0.1 mL of AH extracted via clear cornea paracentesis at the end of surgery for brachytherapy plaque placement. We included 70 AH samples from 70 UM eyes. Molecular results were coded and maintained separately from clinical data and thus did not alter patient treatment for all participants.

### 4.2. Specimen Collection and Storage

A clear corneal paracentesis with a 30-gauge needle was performed to extract ~0.1 mL of AH from UM eyes during clinically indicated surgery to treat UM. The extraction method has been described in detail and published previously by our group for specimen collection from retinoblastoma eyes [19]. Briefly, needles only entered the anterior chamber via the clear cornea at the limbus and did not contact the iris, lens, vitreous, or UM tumor. AH samples were collected intraoperatively but prior to plaque placement or any significant surgical manipulation. Samples were stored on dry ice immediately and transferred to −80 °C within hours for future tests. All samples underwent identical pre-analytic handling and were stored at −80 °C until analysis. Routine FNABs with either a 25- or 27-gauge needle were conducted on 48 patients for mutational analysis and 48 patients for GEP and PRAME status as established clinical tests, which were performed at Castle Biosciences (Phoenix, AZ, USA).

### 4.3. Proximity Extension Assay (PEA) for Protein Quantification

All aqueous humor samples were collected using a standardized protocol at a single center. Samples were processed uniformly, stored at −80 °C immediately after collection, and underwent no more than one freeze–thaw cycle prior to analysis. Protein expression was initially quantified using next-generation sequencing (NGS)-based PEA, which outputs normalized protein expression (NPX) values. NPX values are unitless, log_2_-transformed measures that reflect relative abundance between samples within the same run. While highly reproducible and suitable for comparative analysis, NPX values do not represent absolute protein concentrations and are not directly comparable across experiments or clinical settings.

To enable absolute quantification (AQ), leftover sample volume from 27 AH specimens was used for additional analysis via a qPCR-based PEA platform (Olink, Uppsala, Sweden). This platform yields absolute concentrations (pg/mL) for a defined set of targets. Figure A1 describes the data workflow of these eighty-eight targets. Nine proteins lacked corresponding NPX values in the broader NGS dataset, limiting downstream analysis to 79 proteins. These absolute values served as calibration anchors for extrapolating AQ across the full dataset using regression modeling (Figure A1). Because absolute concentrations were experimentally measured only in this subset, all reported pg/mL values in the full cohort represent calibrated, regression-modeled estimates rather than direct measurements.

### 4.4. Regression-Based Extrapolation of Absolute Concentrations

Linear regression models were constructed for 79 calibration proteins to correlate NPX values from high-throughput PEA with absolute concentrations derived from qPCR-based analysis. The 27 qPCR-assayed samples, including 13 technical duplicates, yielded 40 paired measurements used to construct the calibration models. These models enabled extrapolation of absolute protein concentrations (pg/mL) across the full 70-sample cohort. Inclusion of technical replicates allowed our models to be estimated using real-world variations in NPX values that can be observed across multiple NGS runs from the same source. Because no bridging samples were originally included across the two NGS runs, we performed an additional targeted Olink qPCR-based run that included 40 samples spanning the NPX1-qPCR (2021) and NPX2-qPCR (2023) datasets. This bridging run allowed us to align and correct for batch effects between the two measurement panels. Batch effects were corrected using linear adjustments (median difference between bridging samples analyzed across platforms) and by including batch as a covariate in models. Regression performance showed strong linearity across most proteins (see Figure A4 for R^2^ values and model diagnostics).

### 4.5. Gene Ontology and Pathway Analysis

Functional annotation of protein expression data was performed using QIAGEN’s Ingenuity^®^ Pathway Analysis (IPA^®^, QIAGEN, Hilden, Germany; web-based version: www.qiagen.com/ingenuity, accessed on 20 May 2025). A total of 23 proteins differentially expressed between early-stage (Stages I + II, *n* = 56) and advanced-stage (Stages III + IV, *n* = 10) cases were subjected to canonical pathway and upstream regulator analysis (URA). Protein-level fold changes and statistical significance were assessed using the Mann–Whitney U test, and results were uploaded to IPA for downstream interpretation. IPA generated activation z-scores and corresponding *p*-values for each pathway or regulator. The z-score predicts the activation state of a pathway or regulator, with positive values indicating activation and negative values indicating inhibition. Pathways or regulators with an absolute z-score ≥ 2 were considered significantly activated or inhibited.

### 4.6. Statistical Analysis

All statistical analyses were performed in R version 4.4.1 (R Core Team, 2024). Linear regression models were constructed using the lm() function, with custom scripts for normalization and extrapolation. These models integrated NPX values from two independent NGS-based PEA runs with absolute quantification data obtained from qPCR-based PEA (79 proteins; 40 paired data points across 27 samples, including 13 technical duplicates), enabling extrapolation of absolute protein concentrations (pg/mL) across the full cohort of 70 UM samples. To correct for any heteroscedasticity in model residuals, Huber–White robust standard error terms were used. Multiple testing correction was performed using the Benjamini–Hochberg False Discovery Rate procedure, resulting in 66 proteins retained for downstream analysis (Figure A1). Overlapping samples across the qPCR runs provided internal cross-validation and supported consistency of protein measurements across platforms.

Of the 66 proteins, 43 had median concentrations below 5 pg/mL and were excluded from further analysis due to low abundance, which limits the feasibility of developing analytically robust assays at this concentration range. Differential protein expression was assessed using rank-based statistical tests for each protein when comparing patient groups stratified by GEP/PRAME status or clinical stage (Figure 3A,B). We report untransformed protein concentrations in the figures to preserve interpretability, alongside *p*-values derived from ranked data for more robust significance testing. To assess the robustness of regression-modeled concentrations, a simulation-based sensitivity analysis using prediction intervals was performed; results based on modeled data were compared to *n* = 100 datasets of values that were randomly adjusted to other values within the prediction interval. Data visualization was conducted in R, with boxplots used to illustrate expression distributions across groups. All statistical tests were two-sided, with significance defined as FDR-adjusted *p* < 0.05 unless otherwise indicated.

## 5. Conclusions

This study demonstrates that aqueous humor proteomics, using calibrated protein concentration estimates, is both feasible and clinically informative in uveal melanoma. We established a reference map of protein concentrations and identified a subset of 23 proteins consistently measurable above practical detection thresholds. Many of these showed associations with both molecular risk categories (GEP/PRAME) and clinical stage (AJCC), highlighting their dual diagnostic and prognostic potential. By quantifying proteins in calibrated concentration units, this work provides a reproducible framework for cross-study comparison that may facilitate future assay standardization. These findings should be considered exploratory and not a finalized diagnostic or prognostic classifier; further validation in larger and independent cohorts will be required. Nonetheless, the study lays essential groundwork for developing standardized AH-based biomarker assays and supports the potential of AH as a minimally invasive liquid biopsy platform in uveal melanoma.

## Data Availability

The original contributions presented in this study are included in the article. Due to NIH funding, the data will be available to other researchers via NIH GDS/dbGAP-controlled databases and will also be available to the public upon request from the corresponding author.
